# Enrichment of lung cancer computed tomography collections with AI-derived annotations

**DOI:** 10.1038/s41597-023-02864-y

**Published:** 2024-01-04

**Authors:** Deepa Krishnaswamy, Dennis Bontempi, Vamsi Krishna Thiriveedhi, Davide Punzo, David Clunie, Christopher P. Bridge, Hugo J. W. L. Aerts, Ron Kikinis, Andrey Fedorov

**Affiliations:** 1 Brigham and Women’s Hospital, Boston, MA USA; 2 Artificial Intelligence in Medicine (AIM) Program, Mass General Brigham, Harvard Medical School, Boston, MA USA; 3 Radiology and Nuclear Medicine, CARIM & GROW, Maastricht University, Maastricht, The Netherlands; 4 Radical Imaging, Boston, MA USA; 5 PixelMed Publishing, Bangor, PA USA; 6 Department of Radiology, Massachusetts General Hospital, Boston, MA USA; 7 Department of Radiation Oncology, Brigham and Women’s Hospital, Dana-Farber Cancer Institute, Harvard Medical School, Boston, MA USA

**Keywords:** Lung cancer, Computer science

## Abstract

Public imaging datasets are critical for the development and evaluation of automated tools in cancer imaging. Unfortunately, many do not include annotations or image-derived features, complicating downstream analysis. Artificial intelligence-based annotation tools have been shown to achieve acceptable performance and can be used to automatically annotate large datasets. As part of the effort to enrich public data available within NCI Imaging Data Commons (IDC), here we introduce AI-generated annotations for two collections containing computed tomography images of the chest, NSCLC-Radiomics, and a subset of the National Lung Screening Trial. Using publicly available AI algorithms, we derived volumetric annotations of thoracic organs-at-risk, their corresponding radiomics features, and slice-level annotations of anatomical landmarks and regions. The resulting annotations are publicly available within IDC, where the DICOM format is used to harmonize the data and achieve FAIR (Findable, Accessible, Interoperable, Reusable) data principles. The annotations are accompanied by cloud-enabled notebooks demonstrating their use. This study reinforces the need for large, publicly accessible curated datasets and demonstrates how AI can aid in cancer imaging.

## Background & Summary

National Cancer Institute (NCI) Imaging Data Commons (IDC)^1^ contains publicly available cancer imaging, image-derived, and image-related data, co-located with tools for exploration, visualization, and analysis. Public imaging data contributed by various initiatives, including those from The Cancer Imaging Archive (TCIA)^2^, is ingested into this repository, allowing users to query metadata corresponding to images, annotations, and clinical attributes of the publicly available collections to define relevant cohorts, or subsets, of data. The IDC platform is based on the Google Cloud Platform (GCP), which enables the co-location of data with cloud-based tools for its exploration and analysis. Using tools from GCP, users can form a subset of data (a cohort) that is specific to the task at hand. Users also have the option of creating and using virtual machines to run computationally intensive jobs. Lastly, all analysis steps can be documented using Google Colaboratory python notebooks and shared with others.

Publicly available imaging datasets including the annotation of organs, lesions, and other regions of interest can aid in the development of imaging biomarkers, but unfortunately, many datasets suffer from the limited amount of annotations available. Using IDC, we chose to generate AI annotations for two collections: the Non-small Cell Lung Cancer (NSCLC) Radiomics dataset^2–4^ and a subset of the National Lung Screening Trial (NLST) dataset^2,5,6^. The NSCLC-Radiomics collection contains labeled tumors, and only partially labeled organs of interest (combination of lung, esophagus, heart, and spinal cord). The NLST dataset, though widely used by many researchers, does not contain any image annotations.

In order to annotate the computed tomography (CT) images, we make use of publicly available pre-trained deep learning models for automatically generating annotations. These annotations include volumetric segmentation of organs, the labeling of the region of the body scanned (e.g., chest and abdomen), and landmarks that capture the inferior to superior extent of a selection of organs and bones. The first pre-trained model used is the nnU-Net framework^7^ for volumetric segmentation of thoracic organs. Since the collections we are analyzing concern lung cancer, we chose this model as it produces segmentations of the thoracic organs at risk (heart, aorta, trachea, and esophagus). These regions are routinely used during treatment planning, and could all be affected by the presence of lung cancer (and therefore used to develop new biomarkers or validate published ones). Multiple configurations of the nnU-Net framework (2D vs 3D, low vs high resolution, with and without test-time augmentation) were first applied to the NSCLC-Radiomics collection to evaluate its performance, where the best-performing model configuration was chosen for NLST evaluation. To enhance information about bone and organ landmarks as well as the region of the body, the publicly available body part regression algorithm was employed^8^. The Body Part Regression model^8^ is an unsupervised approach trained on a diverse set of CT data and produces a continuous score for each transverse slice in a 3D volume. These slice scores correspond to specific landmarks obtained from training data, and can then be used to infer the body part region.

To allow for interoperability with the existing tools, we harmonize the representation of the annotations with that of the images being annotated, and implement the Findable, Accessible, Interoperable, and Reuse (FAIR) principles of data curation^9^, we leverage the Digital Imaging and Communications in Medicine (DICOM)^10^ standard. Our dataset is encoded using standard DICOM objects containing volumetric segmentations, slice-level annotations, and segmentation-derived radiomics features. Furthermore, it is accompanied by the complete cloud-ready analysis workflow in the form of Google Colaboratory notebooks that can be used to recreate the dataset, and by the examples demonstrating how to query and visualize those standard objects, and how to convert them into alternative representations.

## Methods

**Fig. 1:**
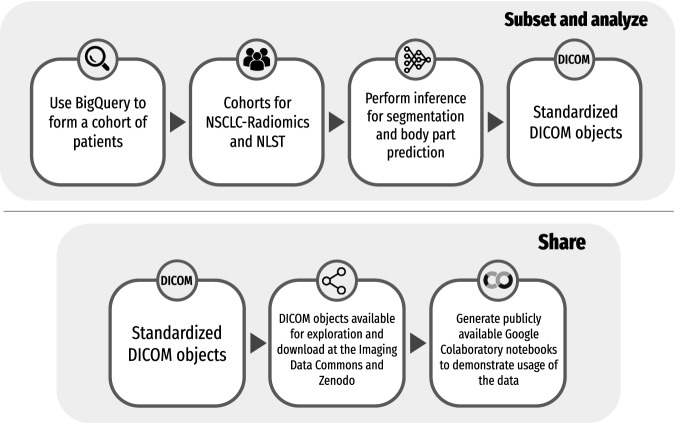
General overview of the study including steps needed to create the DICOM objects and how code and data are publicly shared.

The methods used to perform the preprocessing, analysis, and post-processing of the results are described in detail below. All of the analysis was performed in Google Colaboratory notebooks, making it easier to reproduce our analysis. Fig. 1 gives a general overview of the study and describes the creation of DICOM objects and the sharing of data and code.

### Image collections analyzed

The NSCLC-Radiomics collection^2–4^ is a radiology oncology dataset, where the patient population consists of those with lung cancer. The dataset consists of data from 422 patients scanned at a single institution, as part of the study investigating whether radiomics features can be used to improve cancer detection^3,4^.

The NLST collection^2,5,6^ resulted from the clinical trial that investigated whether low-dose Computed Tomography imaging (CT) could be used to reduce the chance of mortality in a high-risk population of heavy smokers. NLST was a multi-center randomized controlled trial that compared non-contrast, non-ECG-gated low-dose chest CT vs standard chest X-ray imaging for lung cancer screening, where participants were enrolled from 2002 to 2004 and scanned between 2002 and 2007. Participants were included in the study if they were between the ages of 55 and 74 and also smoked more than 30 packs per year, or had quit within the past 15 years. The collection consists of CT images in DICOM format for over 25,000 patients scanned at multiple time points, with a total of over 200,000 DICOM imaging series. Patients from the 33 institutions were scanned using imaging equipment from a variety of vendors and utilizing a range of convolutional kernels.

### Selection of images for analysis

As a prerequisite for the analysis, each CT series must be assembled into a 3D volume. In order to enable such reconstruction, individual slices of the image series must possess consistent attributes that define its geometry, such as the pixel spacing and image orientation, and have no missing slices. Through the use of the IDC platform and integration with GCP, it is possible to filter image series that cannot be reconstructed into a 3D volume using SQL queries interrogating DICOM image metadata, without having to download the image files.

The following criteria were applied to both the NSCLC-Radiomics collection and the NLST collection to select imaging series suitable for the analysis by the AI tools:

1. Consistent orientation of the slices within the series (based on the values of the DICOM ImageOrientationPatient attribute);
2. Consistent in-plane resolution (based on the DICOM PixelSpacing attribute);
3. No overlapping slices within the series (each value of the DICOM ImagePositionPatient within the series must be unique);
4. Consistent (within tolerance) spacing between the adjacent slices within the series (based on the difference in the values of ImagePositionPatient);
5. Series that were identified as localizer, or scout scans based on metadata were excluded (based on the ImageType attribute);
6. A single series was chosen to be analyzed from each study of a selected patient (based on the first SeriesInstanceUID in the list).

Additional selection criteria were applied for the NLST collection:

1. Greater than 100 slices: Some patients may have incomplete scans, and therefore this criterion was introduced to detect potential issues;
2. SliceThickness greater than 1.5 mm and less than 3 mm: we observed the distribution of SliceThickness values for patients in the collection and imposed these criteria in order to remove potential outlier cases;
3. Only patients that screened positive for cancer were considered (this selection criterion was used to select a manageable size of the cohort, since in this study we did not aim to analyze the entire NLST collection due to its large size).

Version 10 of IDC data was used for the query. The queries that were used to perform the filtering of the relevant series are here:

1. NSCLC-Radiomics: https://github.com/ImagingDataCommons/nnU-Net-BPR-annotations/blob/main/common/queries/NSCLC_Radiomics_query.txt
2. NLST: https://github.com/ImagingDataCommons/nnU-Net-BPR-annotations/blob/main/common/queries/NLST_query.txt

After applying the selection criteria, cohorts of 414 patients (414 CT series) and 571 patients (1,039 CT series) were identified for analysis from the NSCLC-Radiomics and NLST collections, respectively. Please refer to Fig. S1 in the supplementary material for an overview of the selection process for both collections in terms of the number of series. Table 1 includes information concerning the number of patients, studies, and series before and after filtering for the two collections. For a list of the series processed for NSCLC-Radiomics and NLST, please refer to https://github.com/ImagingDataCommons/nnU-Net-BPR-annotations/blob/main/common/queries/zenodo_nsclc_radiomics_series_analyzed.csv and https://github.com/ImagingDataCommons/nnU-Net-BPR-annotations/blob/main/common/queries/zenodo_nlst_series_analyzed.csv respectively.

**Table 1:** Characteristics for each analyzed collection before and after applying selection filters.

	NSCLC-Radiomics Entire cohort	NSCLC-Radiomics Analyzed subset	NLST Entire cohort	NLST Analyzed subset
Number of patients	422	414	26,254	571
Number of studies	422	414	73,113	1,039
Number of series	422	414	203,087	1,039

For processing the cohort, the queries were run once. The Unique Resource Identifiers (URIs) corresponding to the files of the selected series were used to retrieve the files from the IDC Google Storage buckets.

### Preprocessing and processing steps

**Fig. 2:**
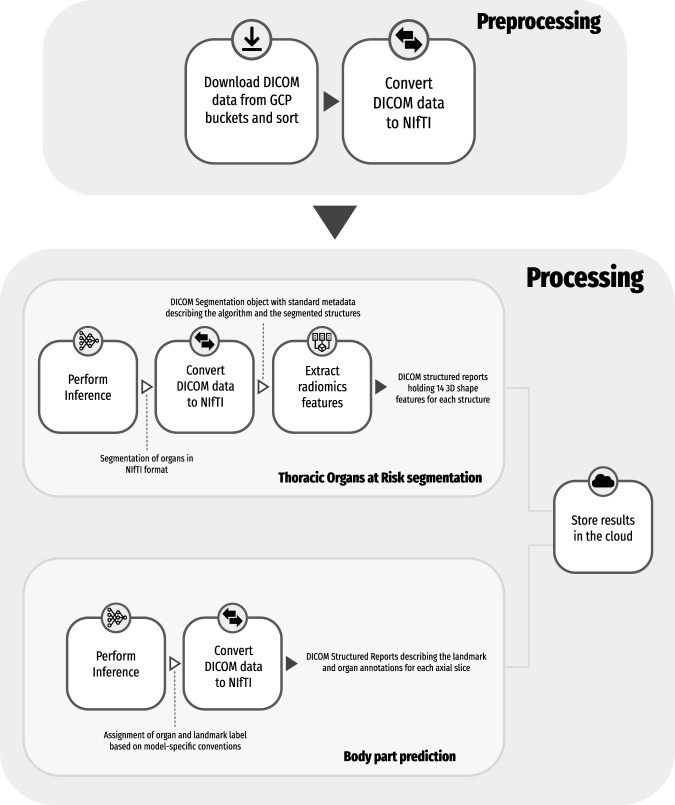
Processing steps for both segmentation of organs and body part landmark and region annotation.

Fig. 2 gives an overview of the preprocessing and processing steps in the pipeline. The DICOM files were downloaded from publicly hosted GCP buckets and sorted using the *dicomsort* package (https://github.com/dicomsort/dicomsort). The package *dcm2niix*^11^ (https://github.com/rordenlab/dcm2niix) was used to perform the conversion of DICOM to the NifTI format required by the analysis tools. Three of the series that passed the BigQuery checks had inconsistent attributes that were not considered in the query, leading to failure to convert into NifTI format, and were subsequently discarded. The processing pipeline consists of two streams: (1) volumetric segmentation of the regions of interest; and (2) anatomic region and landmark annotation. Inference was performed using a pre-trained model for both use cases. Radiomics shape features were computed from the volumetric segmentations of the regions of interest. Finally, DICOM Segmentation and Structured Report objects were created for archival representation of the obtained analysis results.

### Anatomic region segmentation pipeline

#### Inference using a pre-trained model

The nnU-Net deep learning framework^7^ was used for the automatic segmentation of the heart, aorta, trachea, and esophagus. The nnU-Net framework introduced a data-driven heuristic for hyperparameter selection that has proven to be very efficient for small datasets, yielding models that performed well in a number of challenges, including The Medical Segmentation Decathalon^12^ and AMOS 2022^13^. These pre-trained models, developed for different imaging modalities and various body regions were shared with the original publication. nnU-Net performance and robustness in the aforementioned challenges made it a de-facto popular baseline for benchmarking other deep learning models. The Task055 nnU-Net model we used is part of the collection of pre-trained models and was trained on data from the SegTHOR challenge^14^, where the goal was to delineate thoracic organs of interest for the purposes of radiotherapy.

The authors of nnU-Net provided a python package (https://github.com/MIC-DKFZ/nnUNet) (v1) to run training, evaluation, and inference. The implementation includes a command-line tool to easily run inference using a pre-trained model. The pre-trained model for Task055 SegTHOR was downloaded from Zenodo^15^. nnU-Net provides as output a single NifTI file containing the segmented structure (label 1 = esophagus, label 2 = heart, label 3 = trachea, label 4 = aorta).

#### Radiomics feature extraction

The *pyradiomics* package^16^ (version v3.0.1) was employed to extract 3D shape features from the regions segmented by nnU-Net. The package is an open-source library of functions required to extract a variety of radiomic features from a set of regions. The following features were extracted:

1. Elongation
2. Flatness
3. Least Axis Length
4. Major Axis Length
5. Maximum 3D Diameter
6. Mesh Volume
7. Minor Axis Length
8. Sphericity
9. Surface Area
10. Surface Volume Ratio
11. Voxel Volume
12. Compactness 1
13. Compactness 2
14. Spherical Disproportion

### Body part landmark and region annotation pipeline

#### Inference using a pre-trained model

The Body Part Regression^8^ is an unsupervised model developed to provide slice-level annotations of the presence of anatomical landmarks for axial CT volumes. The locations of the landmarks are then used to infer which parts of the body each axial slice corresponds to. There are numerous advantages to using such a method – the method does not rely on segmentations to train the neural network, thereby affording ease of use in case of retraining. The method produces not only annotations of organs identified in each axial slice but also a set of landmarks that can assist in further downstream applications. The pre-trained model performs localization of 35 bone and organ landmarks, including the cervical, thoracic, and lumbar vertebrae. Additionally, six body part regions are defined from the landmarks including the head, shoulder-neck, chest, abdomen, pelvis, and legs.

The authors provided a python package^17^ (also available at https://github.com/MIC-DKFZ/BodyPartRegression) to perform inference using a pre-trained model. The pre-trained model was downloaded from Zenodo^18^ (version 1.1). The output of the model provides a JSON file that holds the values of the slice scores, the corresponding landmark lookup table, and the slice indices that correspond to each body part region.

**Fig. 3:**
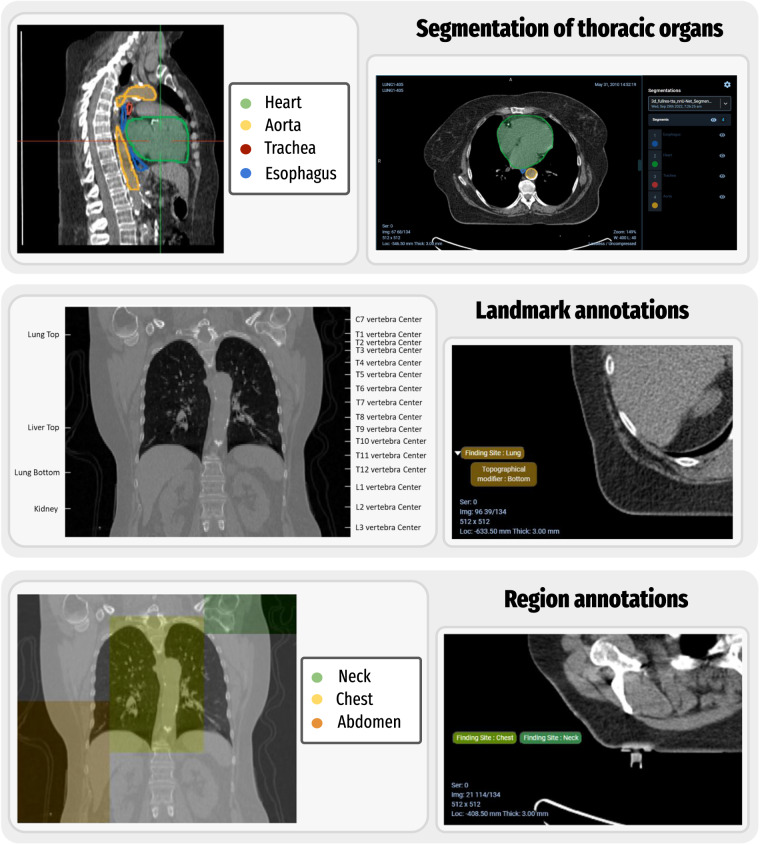
Examples of automatically generated annotations (top) nnU-Net segmentation examples showing a multi-planar reconstruction view (middle) landmark annotation example for a series (bottom) region annotation example for a series.

Fig. 3 gives examples of the automatically generated annotations including the thoracic organ segmentations from nnU-Net, and the body part prediction landmarks and regions.

## Data Records

To ensure FAIR representation of data and metadata, all of the analysis results produced were converted into DICOM representation. DICOM standard is the international standard for medical images and related information^10^. It includes capabilities to describe, encode, and exchange results of image analysis, such as those produced in the process of our study.

For the NSCLC-Radiomics collection, the following are included for each of the cases analyzed:

1. Three Segmentation objects that hold the nnU-Net predictions for 2d + tta, 3d_lowres + tta, and 3d_highres + tta, respectively
2. Three Structured Reports that hold the radiomics shape feature computations for 2d + tta, 3d_lowres + tta, and 3d_highres + tta models respectively
3. A Structured Report that holds the body part landmarks
4. A Structured Report that holds the body part regions

For the NLST collection, the following are included:

1. A Segmentation object that holds the nnU-Net predictions for the 3d_highres + tta model
2. A Structured Report that holds the radiomics shape feature computations for the 3d_highres + tta model
3. A Structured Report that holds the body part landmarks
4. A Structured Report that holds the body part regions

All of the data that are described here are available as part of the Zenodo “AI-derived annotations for the NLST and NSCLC-Radiomics computed tomography imaging collections”^19^. The data is also available within IDC as of version v13^20^ (https://portal.imaging.datacommons.cancer.gov/explore/filters/?analysis_results_id=nnU-Net-BPR-annotations). As the data followed the standard DICOM representation, it was possible to store the data in a Google Healthcare DICOM datastore and automatically extract the metadata. This allows for the data to then be searchable and queried easily. Once stored in the DICOM datastore, it was then possible to visualize images and corresponding volumetric and slice-level annotations using the OHIF DICOM viewer^21^ integrated with IDC Portal (https://portal.imaging.datacommons.cancer.gov/). We further demonstrate those features in the subsequent sections and the accompanying materials.

### Segmentations

Each label map was first saved as a separate NifTI file (.nii.gz). The *itkimage2segimage* tool from the software package *dcmqi*^22^ (version 1.2.5) was used to convert the segments from the nnU-Net output to a DICOM Segmentation (SEG) object. The annotations for the four thoracic organs for each of the analyzed CT series were saved as a single SEG object. Each SEG object contains a list of the ReferencedSOPInstanceUIDs within the ReferencedSeriesSequence that refer back to the original CT slices. Segment-level DICOM attributes document the type of the algorithm used, and coded terms describing the content of the segment. Table 2 is used for the creation of the JSON metadata file needed for conversion to a SEG object. The table is also available here [https://github.com/ImagingDataCommons/nnU-Net-BPR-annotations/blob/main/nnunet/data/nnunet_segments_code_mapping.csv].

**Table 2:** The segments code mapping file used to create a JSON metadata file for conversion of NIfTI files to DICOM Segmentation objects.

Segment	Finding CodingSchemeDesignator	Finding CodeValue	Finding CodeMeaning	FindingSite CodingSchemeDesignator	FindingSite CodeValue	FindingSite CodeMeaning
Esophagus	SCT	113343008	Organ	SCT	32849002	Esophagus
Heart	SCT	113343008	Organ	SCT	80891009	Heart
Trachea	SCT	113343008	Organ	SCT	44567001	Trachea
Aorta	SCT	113343008	Organ	SCT	15825003	Aorta

### Radiomics features

The 3D shape radiomics features were saved as Enhanced Structured Reports (SR) with the TID1500 template using the command *tid1500writer* from the *dcmqi*^22^ package (version 1.2.5). A single SR was saved for each series analyzed. Each feature is defined in the IBSI standard^23^ and is described by the coded quantity and units. Table 3 was used for the creation of the DICOM Structured Reports for holding radiomics features. The table is also available here [https://github.com/ImagingDataCommons/nnU-Net-BPR-annotations/blob/main/nnunet/data/nnunet_shape_features_code_mapping.csv]. Overall, 14 3D shape features were chosen for the analysis.

**Table 3:** The shape features code mapping file used to create the TID1500 DICOM Structured Reports.

shape_feature	quantity_Coding Scheme Designator	quantity_CodeValue	quantity_CodeMeaning	units_Coding Scheme Designator	units_CodeValue	units_CodeMeaning
Elongation	IBSI	Q3CK	Elongation	UCUM	1	no units
Flatness	IBSI	N17B	Flatness	UCUM	1	no units
LeastAxis Length	IBSI	7J51	Least Axis in 3D Length	UCUM	mm	millimeter
MajorAxis Length	IBSI	TDIC	Major Axis in 3D Length	UCUM	mm	millimeter
Maximum3D Diameter	IBSI	L0JK	Maximum 3D Diameter of a Mesh	UCUM	mm	millimeter
MeshVolume	IBSI	RNU0	Volume of Mesh	UCUM	mm3	cubic millimeter
MinorAxis Length	IBSI	P9VJ	Minor Axis in 3D Length	UCUM	mm	millimeter
Sphericity	IBSI	QCFX	Sphericity	UCUM	1	no units
SurfaceArea	IBSI	C0JK	Surface Area of Mesh	UCUM	mm2	square millimeter
SurfaceVolumeRatio	IBSI	2PR5	Surface to Volume Ratio	UCUM	/mm	per millimeter
VoxelVolume	IBSI	YEKZ	Volume from Voxel Summation	UCUM	mm3	cubic millimeter
Compactness1	IBSI	SKGS	Compactness 1	UCUM	1	no units
Compactness2	IBSI	BQWJ	Compactness 2	UCUM	1	no units
Spherical Disproportion	IBSI	KRCK	Spherical Disproportion	UCUM	1	no units

### Body part regression landmarks

The landmarks produced from the body part prediction neural network were saved as Comprehensive 3DSR DICOM SRs, where a single report was saved for each series analyzed, and individual landmarks were associated with the image slices through the references to the corresponding SOPInstanceUIDs. The *highdicom* package^24^ (version 0.20.0) was used to generate the SRs. Please refer to Table S1 in the supplementary material for the metadata concerning the mapping of each of the body part regression codes to specific bones and landmarks. The table is also available here [https://github.com/ImagingDataCommons/nnU-Net-BPR-annotations/blob/main/bpr/data/bpr_landmarks_code_mapping.csv].

### Body part regression regions

Inference by the body part prediction neural network also includes a region(s) assignment for each axial slice. This region assignment information was saved as DICOM SRs, where a single report was saved for each series analyzed, and body part assignment was performed at the slice level via the referenced SOPInstanceUID. The *highdicom* package^24^ (version 0.20.0) was used to generate the SR. Table 4 holds the mapping of each body part region to specific target regions and is also available here [https://github.com/ImagingDataCommons/nnU-Net-BPR-annotations/blob/main/bpr/data/bpr_regions_code_mapping.csv].

**Table 4:** The region code mapping file used to create the TID1500 DICOM Structured Reports.

BPR_code_region	CodingSchemeDesignator	CodeValue	CodeMeaning
legs	SCT	30021000	Legs
pelvis	SCT	12921003	Pelvis
abdomen	SCT	113345001	Abdomen
chest	SCT	51185008	Chest
shoulder-neck	SCT	45048000	Neck
head	SCT	69536005	Head

## Technical Validation

In the following sections, we summarize the results of the evaluation. All of the results are available for interactive exploration using the Google Colaboratory notebooks accompanying the publication.

### Image preprocessing

The Methods section concerning the selection of images for the analysis described the series of filters that were used to create a curated dataset for both the NSCLC-Radiomics and NLST collections. Results of the query were stored in a BigQuery table, to ensure the same series were used for the subsequent steps of the analysis.

As described previously, the CT DICOM files were converted to the NifTI format, as this format was used as input for both the nnU-Net prediction as well as the body part regression method. The package *dcm2niix*^11^ (https://github.com/rordenlab/dcm2niix) version 1.0.20220720 was used to perform the conversion. If multiple NifTI volumes were created from the conversion, the file with the number of slices matching the number of DICOM files was chosen. Further processing of the series was skipped if other errors were produced by *dcm2niix*^11^.

### DICOM object validation

Before analyzing the results in the DICOM Segmentation objects and Structured Reports, they were validated using publicly available tools. To check the DICOM conformance of the objects, the tool *dciodvfy* from *dicom3tools (*https://www.dclunie.com/dicom3tools.html) was used. *dicom3tools* is a set of command line utilities that can create, validate, and modify DICOM files, and also perform conversion to DICOM. For the SR objects, the software package *DICOMSRValidator* from *PixelMed* (http://www.pixelmed.com/index.html#PixelMedJavaDICOMToolkit) was used to check conformance with the TID1500 DICOM standard SR template.

### Segmentation analysis

#### Comparison of the NSCLC-Radiomics expert segmentations to the AI-derived segmentations

**Fig. 4:**
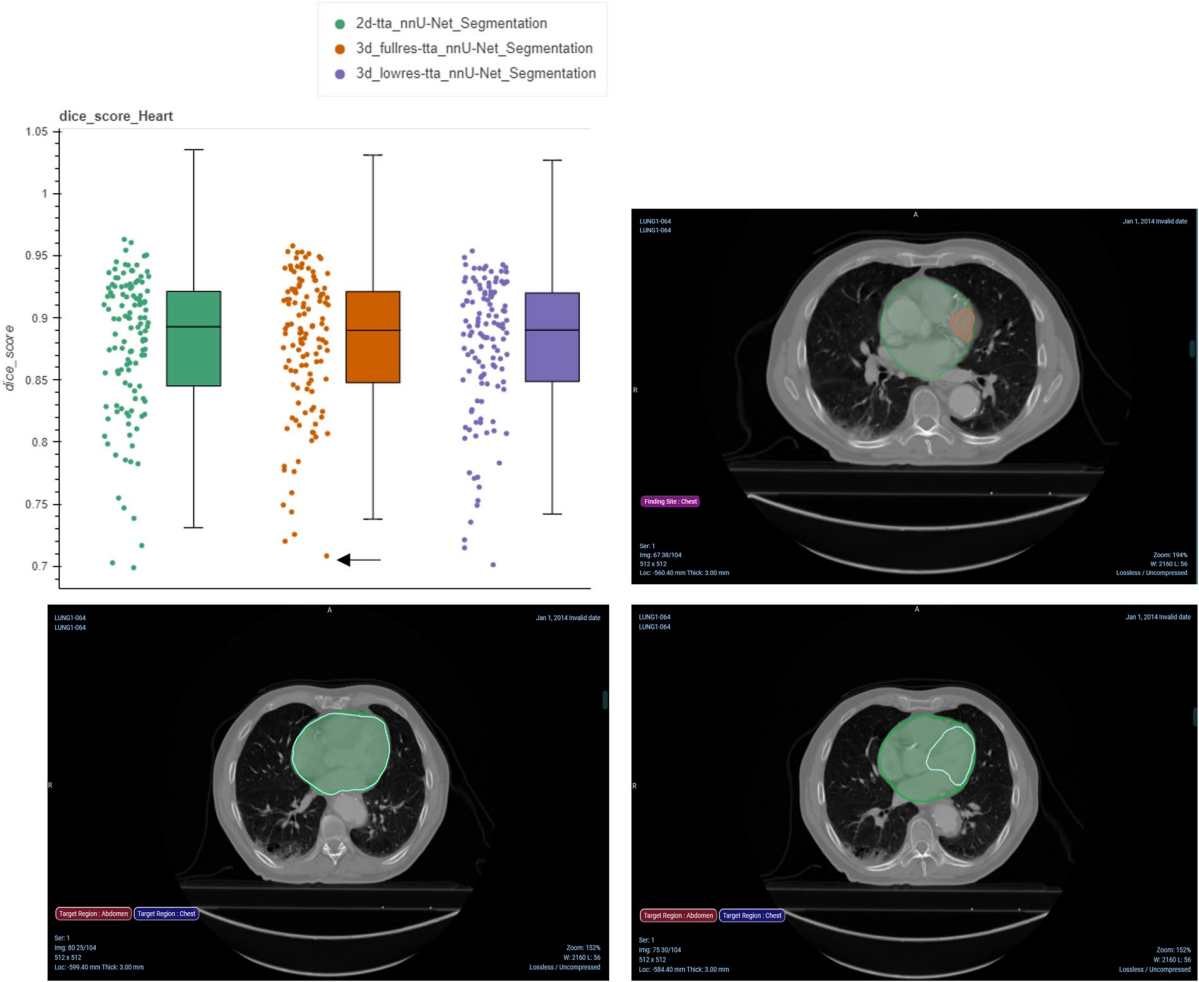
Evaluation of the AI-generated annotations with respect to the expert annotations of the heart for NSCLC-Radiomics. Top left: Dice score computation between each AI-generated annotation and the expert segmentation, Top right: point (highlighted by the black arrow) in the left pane corresponds to the visualization of the analysis results in the OHIF Viewer, with the ground truth in red and the AI-derived segmentation in green. Bottom left: Qualitative example of the nnU-Net model prediction in green to the ground truth in white with high overlap, Bottom right: Qualitative example of the nnU-Net model prediction in green to the ground truth in white with low overlap.

A subset of the series from the NSCLC-Radiomics collection contains expert segmentations of the heart and the esophagus. Therefore, it was possible to quantitatively assess the results by evaluating the Dice score and Hausdorff distance between the expert- and AI-derived segmentations. Fig. 4 displays the Dice score metric of the heart for the three nnU-Net models evaluated, where in general, high overlap results are demonstrated with all three AI-derived models. Interactive notebooks and plots accompanying this manuscript allow picking a point with the lowest Dice score for the 3D full resolution and test time augmentation model to visualize images and annotations using the OHIF viewer^21^. The differences between the expert and AI-derived annotations may be due to variations in the criteria used to delineate the heart between the expert and the data used for the pre-trained nnU-Net model. Segmentations for the NSCLC-Radiomics collection were provided by a single radiation oncologist using manual contouring on 2D slices^3^ whereas for SegTHOR^14^ manual annotations were provided by a radiation oncologist following the criteria recommended by the Radiation Therapy Oncology Group 2^14^.

#### Using shape radiomics features for outlier detection within AI segmentations for the NSCLC-Radiomics collection

**Fig. 5:**
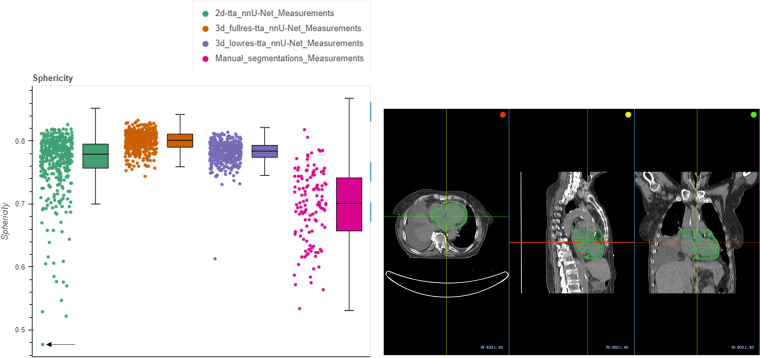
Evaluation of the heart sphericity radiomics features from the AI-generated annotations compared to the expert from NSCLC-Radiomics. Left: Radiomics distribution computed from each AI-generated annotation and expert annotation, and Right: point (highlighted by the black arrow) in the left pane corresponds to the visualization of the analysis results in the OHIF viewer, for the lowest sphericity value.

Another way to detect outliers in the segmentations apart from the Dice score and Hausdorff distance metrics is to perform a radiomics analysis, where features with high variance or with outliers may point to problematic analysis results. We compared the radiomics features extracted from the three AI-derived models to the features from the expert segmentations for NSCLC-Radiomics. Fig. 5 displays this for the sphericity feature of the heart, where values closer to 1 indicate higher sphericity. We can see that the 3D full resolution model displays a narrow distribution of high sphericity values compared to the 3D low resolution and the 2D resolution models, which are expected as the 3D full resolution should ideally perform more accurately for our volumetric data. We can also see that the expert segmentations have a wide distribution, which may be due to inconsistencies with the delineation, as it was performed slice-wise. Picking the series with the lowest value of sphericity (left) yields the 2D multi-planar reconstruction (MPR) view in OHIF, where as expected, it can be seen that the 2D model does not perform well for the series.

#### Using shape radiomics features for outlier detection within AI segmentations for the NLST collection

**Fig. 6:**
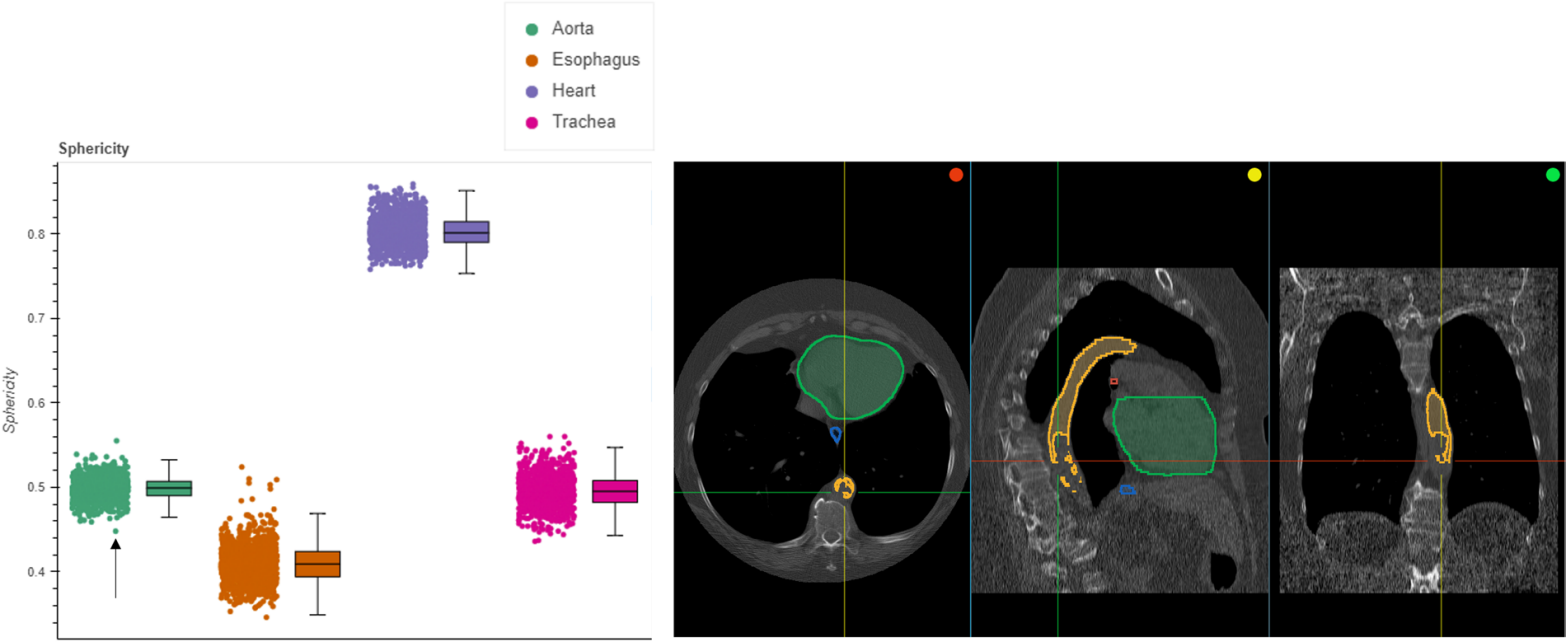
Evaluation of the sphericity radiomics features from the AI-generated annotations from NLST. Left: Radiomics distribution computed for the trachea, esophagus, heart, and aorta, and Right: point (highlighted by the black arrow) in the left pane corresponds to the visualization of the analysis results in the OHIF viewer, for the lowest value of the aorta.

Unlike the NSCLC-Radiomics collection, the NLST collection does not contain ground truth segmentations. Therefore, the analysis of the radiomics features for outlier detection was relied upon. Fig. 6 demonstrates the distributions of sphericity values for the four segmented regions. We can see that though in general, the points are within the whisker ranges, but some go beyond, for instance in the aorta. Picking the point with the lowest sphericity value for the aorta yields the case where it has not been properly segmented by the AI model. This could be due either to the performance of the pre-trained model not being able to generalize to new data, or due to abnormalities in the patient.

### Landmark analysis

#### Validation of the lung start and end landmarks using the ground truth lung segmentation for NSCLC-Radiomics

**Fig. 7:**
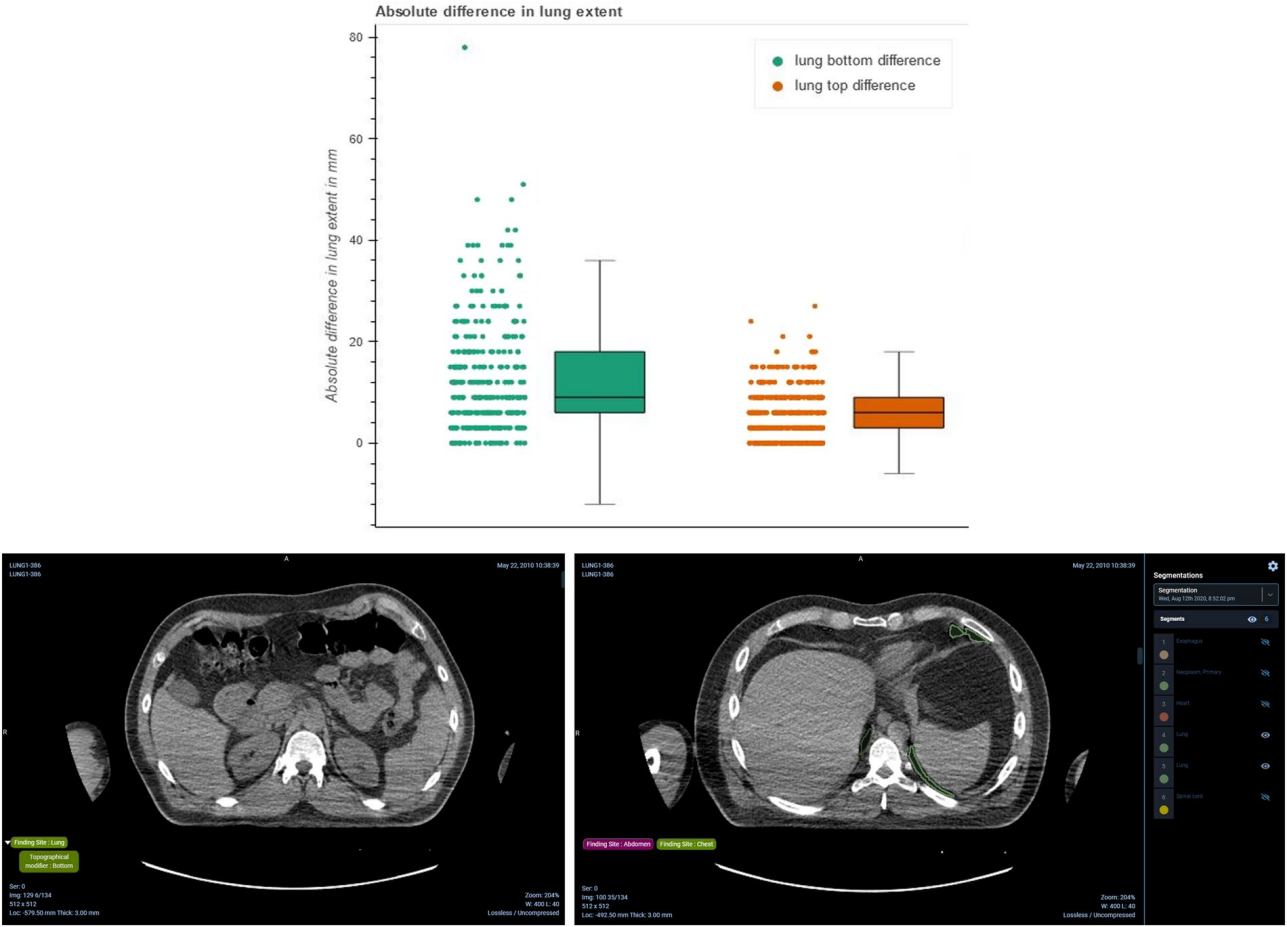
Demonstration of the difference between the body part regression predicted landmarks and the expert lung segmentations. Top: Boxplot displaying the difference between the most inferior point of the ground truth lung to the lung_start landmark, and the difference between the most superior point of the ground truth lung to the lung_end landmark, and Bottom: Qualitative results in the OHIF viewer displaying the difference between the predicted landmark axial slice (left) and the most inferior slice of the expert segmentation (right).

To assess the accuracy of the body part predicted landmarks, we evaluate their locations against the expert segmentations. For NSCLC-Radiomics, the segmentation of the left and right lungs is provided for a majority of the patients. Therefore we compared the bottom of the lung and top of the lung landmarks to the inferior and superior axial slice locations of the expert segmentations. The top panel in Figure 7 displays the distributions of these two sets of differences, where the ‘lung bottom difference’ represents the difference between the bottom of the lung landmark and the inferior axial slice location of the expert segmentation, and the ‘lung top difference’ represents the difference between the top of the lung landmark and the superior axial slice location of the expert. The bottom panel in Figure 7 demonstrates an example of disagreement between the body part predicted slice and the expert, where the predicted landmark is more inferior. This could be due to abnormalities in the anatomy of the patient.

#### Calculation of the lung extent

**Fig. 8:**
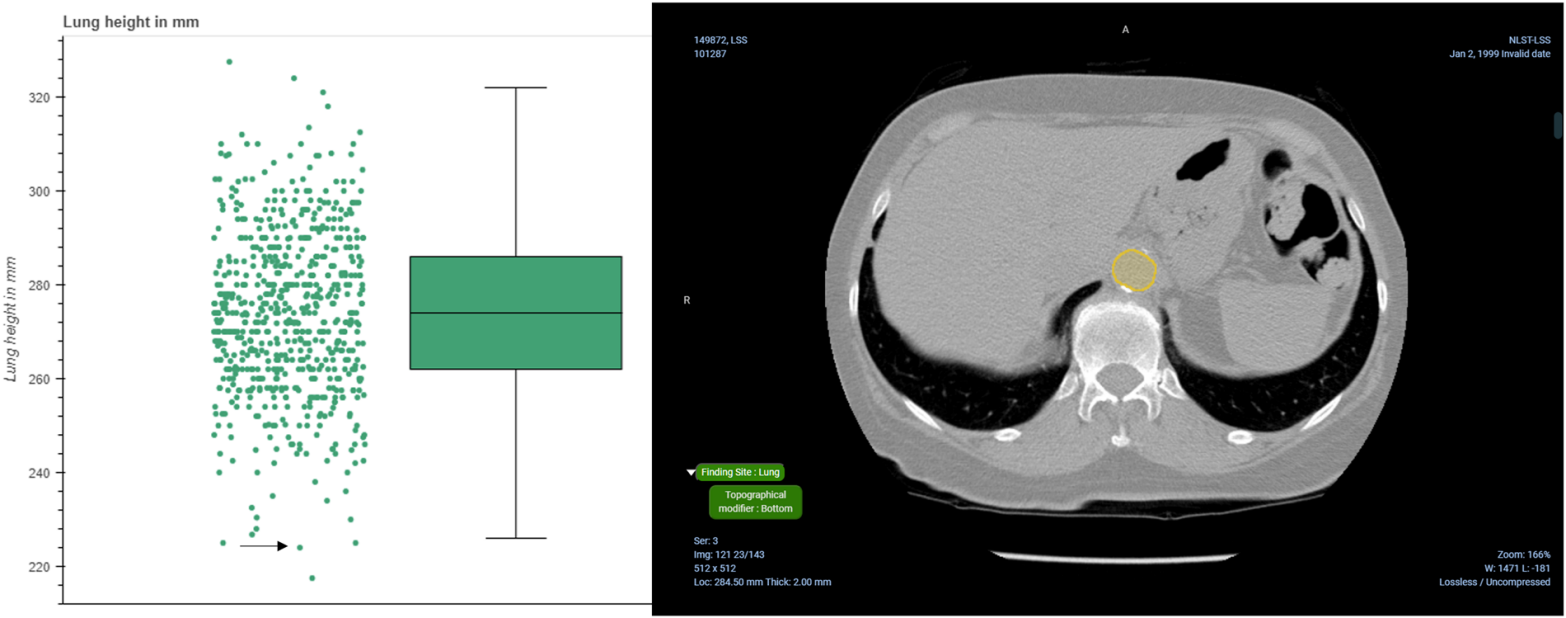
Evaluation of the distribution of distances between the start and end of the lungs in mm for the NLST collection. Left: Distribution of distances between the start and end of the lungs, and Right: point (highlighted by the black arrow) in the left pane corresponds to the visualization of the analysis results in the OHIF viewer, where the identification of the lung start slice is slightly off.

In the absence of manual annotations for the NLST collections, we resort to the analysis of the distributions of various measures we can calculate from the produced annotations. One such measure is lung height, which we calculated the distance between the *lung_start* and *lung_end* landmarks in mm. These correspond to the most inferior and most superior axial slices of the lung respectively. Fig. 8 summarizes these height values for the NLST collection, where it can be seen that the median heights are relatively close to the expected height of the lungs in adults, which is 27 cm^25^. Lung heights that are significantly different from the median could indicate an issue with scanning the patient, for instance, an incomplete scan, or it could indicate an anatomical problem with the lung itself. Fig. 8 shows an example of where the identification of the start slice of the lung is slightly off.

### Anatomic region analysis

#### Calculation of the ratio of slices assigned to each body part region

**Fig. 9:**
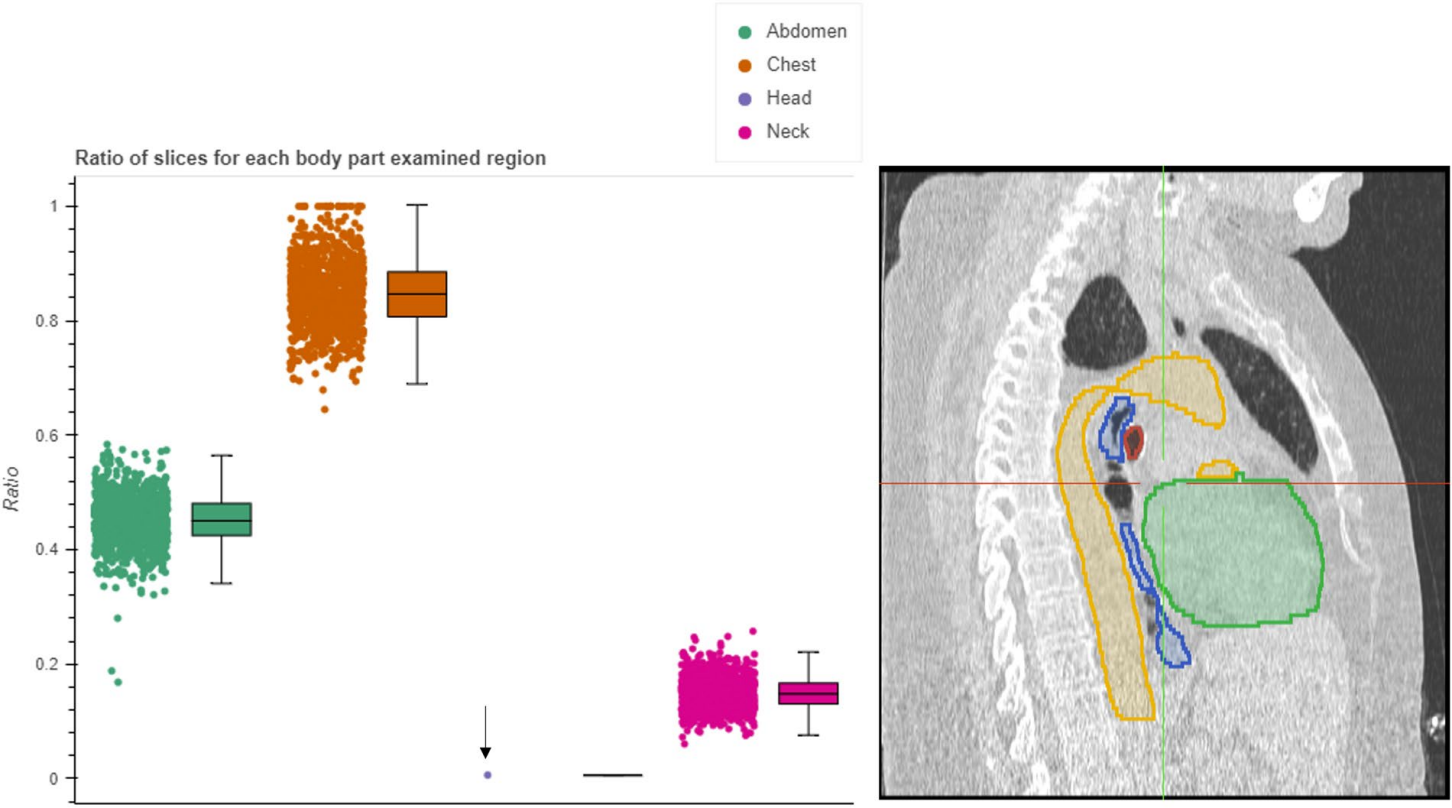
Evaluation of the percentage of slices assigned to each region (head, neck, chest, abdomen, pelvis, and legs) for the NLST collection. Left: Distribution of the percentages for each region, and Right: point (highlighted by the black arrow) in the left pane corresponds to the visualization of the analysis results in the OHIF viewer, where we can confirm the scan includes a portion of the head.

The body part prediction method provides an assignment of regions to each axial slice, where more than one region may be assigned to a slice. To quantitatively assess the assignment of these regions and summarize it over the entire collection, we plotted the ratio of the slices assigned to each body part region. Using this plot one could ensure that the largest proportion of slices was assigned to the chest region, which is expected, since the collections are focused on patients with lung cancer. Fig. 9 displays these slice ratios for the NLST collection, where it can be seen that areas outside of the chest are scanned. We can visually confirm the presence of a portion of the head in the scan.

## Usage Notes

The DICOM Segmentation objects and Structured Reports are available in IDC since data release v13^20^. They are also available in Zenodo^19^ (version 4) at the following link: https://zenodo.org/records/7975081.

There are numerous downstream applications in analyzing data from the NLST and NSCLC-Radiomics collections that may potentially benefit from the availability of anatomical region segmentations, radiomics extraction from those regions, the localization of bone and organ landmarks, as well as the labeling of regions:

- Segmentation of the anatomic structures is a common preprocessing step in image analysis pipelines (e.g., evaluation of cardiovascular risk using quantitative analysis of calcium relies on the segmentation of the heart as a prerequisite^26^, or quantification of airway obstruction/emphysema relies on the segmentation of airways including the trachea^27^).
- Segmentation of thoracic structures is necessary for the identification of organs-at-risk for radiotherapy treatment planning^14^.
- Segmentations generated from the multiple nnU-Net models enable evaluation of the generalizability of the algorithm on an external dataset, and establish a baseline for evaluation of alternative similar algorithms. It becomes straightforward to evaluate the results produced by the nnU-Net segmentation, which can be considered state-of-the-art, and identify its weaknesses to motivate further improvement.
- Shape radiomics features can be used to detect potential outliers in the segmentations, but also can be utilized as quantitative features to stratify patients within the cohort or contribute to applications that utilize such features in more complex analyses.
- Annotations of landmarks and regions can be used to assist in defining regions of interest to simplify the task of segmentation of those structures.
- Given slice-level annotations of the landmarks and anatomic regions, it becomes possible to define anatomy-based search filters. As an example, one can select image volumes that contain specific vertebrae levels, or those that contain abdominal organs or head/neck, within a collection that focuses on chest imaging.

While the algorithms we used are publicly available for anyone to apply to the images we analyzed, the development of the reproducible analysis workflows requires significant effort. Filtering of the images suitable for processing requires an understanding of DICOM data representation and the intricacies of developing selection queries. Inference pipeline computation takes time, bears cost in terms of resources used for the computation and, when using cloud resources, requires an understanding of how cloud resources can be used cost- and time-efficiently. Therefore, we believe there is a significant benefit in providing the resulting analysis artifacts to the community to enable the aforementioned applications.

To demonstrate some of the above applications, we have provided a Google Colaboratory notebook for interaction for both the nnU-Net prediction analysis (including radiomics analysis), along with the body part prediction results analysis: https://github.com/ImagingDataCommons/nnU-Net-BPR-annotations/blob/main/usage_notebooks/scientific_data_paper_usage_notes.ipynb. To perform additional exploratory analyses of the enriched collections, the Google LookerStudio dashboard can be utilized: https://lookerstudio.google.com/s/rWMFI0K4dDM.

For the nnU-Net prediction segmentation analysis, we divide the notebook into the following sections:

1. *Examples of interactive plots comparing expert segmentations vs AI-derived segmentations*.
  The NSCLC-Radiomics collection contains series that have some annotated organs (heart and esophagus), while the NLST collection does not. To choose the best pre-trained nnU-Net model, we chose three models and compared performance to the expert annotations. We then chose the most robust and high-performing model and used that for the NLST collection. Therefore to prove our choice of model, for the NSCLC-Radiomics series with expert annotations, we quantitatively compared the performance of the three nnU-Net models to the expert delineations in terms of Dice and Hausdorff distance metrics. The user can interact with the plots and visualize corresponding images and analysis results in the OHIF viewer.
2. *Examples of how to query and download DICOM Segmentation objects and the associated CT files*.
  Users may want to understand how to query for DICOM Segmentation objects, to perhaps load in 3DSlicer^28^, other external programs, or use locally. We therefore provide code with concrete examples of how to use BigQuery for querying for appropriate data and how to download the files from the public IDC buckets.
3. *Demonstration of how to convert DICOM files, for both the CT files and the segmentation objects using multiple packages*.
  As many AI and ML pipelines require numpy arrays or NIfTI files for holding the segmentation output, we demonstrate how to convert the DICOM Segmentation objects to NIfTI. We use three robust software packages, *dcmqi*^22^, *pydicom_seg* (https://github.com/razorx89/pydicom-seg), and *highdicom*^24^ to demonstrate multiple ways of performing this conversion. *dcmqi* for instance converts a DICOM Segmentation to many research formats and also outputs a JSON file that holds the metadata of the DICOM Segmentation object, in particular the CodingSchemeDesignators, CodeValues, and CodeMeanings necessary to interpret the anatomical object. 3DSlicer^28^ can be used to view the DICOM Segmentation objects, which internally uses *dcmqi* for the conversion to labelmaps. We also show how to convert CT DICOM files to NIfTI using *dcm2niix*^11^ (https://github.com/rordenlab/dcm2niix).
4. *Visualization of DICOM and NIfTI files using ITKWidgets and custom code*.

Instead of downloading the DICOM and NIfTI files (both CT files and the segmentation overlays) and viewing them in external programs, the reader may want to quickly view them within the notebook itself. We make use of *ITKWidgets* (https://github.com/InsightSoftwareConsortium/itkwidgets) and custom code for these demonstrations.

For the nnU-Net radiomics feature evaluation, we demonstrate the following:

1. *Examples of how to query using the radiomics features*.
  The radiomics features are stored in the quantitative_measurements publicly accessible table. We show how querying the metadata is beneficial, instead of manually reading in a set of DICOM SRs and extracting the nested metadata yourself. We demonstrate how to extract radiomics features for a single series, or over all series in the collection, and also how to obtain a list of series where a given radiomics feature falls within a certain range of values.
2. *Examples of interactive plots with the radiomics features values*.

We demonstrate an interactive plot showing the distribution of a single feature for all series, a single region for NSCLC-Radiomics, and a single feature for all series across all regions for NLST. These types of plots can be used to identify possible outliers in the segmentation. The user can also click on, for instance, sphericity feature values, which opens up an OHIF viewer link to examine specific series.

For the Body Part Regression analysis of landmarks, we demonstrate the following:

1. *Plot the location of the landmarks on a coronal slice*.
  Though the landmarks per transverse slice can be visualized in OHIF, it is useful to get a quick assessment of the landmark locations for a patient. We therefore demonstrate the automatic ability to assess the transverse locations of the landmarks on a coronal slice (as demonstrated in Fig. 3 of this paper).
2. *Evaluation of the lung landmark compared to the expert segmentations*.
  Validation of the landmarks in a quantitative manner and not only qualitative is crucial, especially for the NSCLC-Radiomics dataset where expert lung segmentations are available. Therefore we compute the distance between the *lung_top* landmark with the superior point of the expert lung, and the *lung_bottom* landmark with the inferior point of the expert lung segmentation. The user can also interact with this plot and assess if there are any series where the body part prediction is incorrect.
3. *Evaluation of the lung landmark distribution*.
  As the NLST collection does not have expert segmentations, one method to assess the body part prediction is to calculate the distance between the top and bottom lung landmarks and assess for outliers. Again, we demonstrate this using an interactive plot.
4. *Extract series that have specific landmarks*.
  We demonstrate the usefulness of having the metadata extracted from the landmarks SRs in a table, as we can infer which patients do not have certain expected landmarks (e.g., lung).
5. *Download and extract values from the SRs*.

Though the metadata from the landmarks can be made available in a table, the user still has the option of downloading the files and extracting the values. We demonstrate how to perform such an extraction using *dcmqi*^22^ and *highdicom*^24^.

For the Body Part Regression analysis of regions, we demonstrate the following use cases:

1. *Qualitative evaluation of the regions*.
  Though one can view the body part assigned regions in OHIF, one may want a quick overview of the transverse slices assigned to each region. We demonstrate how to do this using *matplotlib*.
2. *Creation of an interactive plot for series that include the chest*.
  We may be interested to know the percentage of slices that are assigned to the chest region. By analyzing these range of values we can see the extent of a scan and detect any outliers.
3. *Creation of an interactive plot for percentages of regions for all series*.
  We may also be interested in quickly assessing differences in scan regions, by seeing if patients have the head, neck, pelvis, or legs included. For instance, we may have included patients with brain scans and would want to filter those scans from our analysis.
4. *Download and extract values from the SRs*.

Though the metadata from the regions can be made available in a table, the user still has the option of downloading the files and extracting the values. We demonstrate how to perform such extraction using *dcmqi*^22^ and *highdicom*^24^.

### Supplementary information

Supplementary Information

## Data Availability

The code for creating the annotations and demonstrating interactions with the data is available as Release v2.0.0^29^, and is also available at https://github.com/ImagingDataCommons/nnU-Net-BPR-annotations/releases/tag/v2.0.0. The Colaboratory usage notebook is available here: https://github.com/ImagingDataCommons/nnU-Net-BPR-annotations/tree/main/usage_notebooks. In order to run the notebook, users must set up a GCP project by following the instructions here https://learn.canceridc.dev/introduction/getting-started-with-gcp. The notebooks used to query, run the analysis, and create the DICOM Segmentation objects and Structured Reports are listed here: 1. NSCLC-Radiomics analysis: https://github.com/ImagingDataCommons/nnU-Net-BPR-annotations/blob/main/nnunet/notebooks/idc_nsclc_nnunet_and_bpr_infer.ipynb. 2. NLST analysis: https://github.com/ImagingDataCommons/nnU-Net-BPR-annotations/blob/main/nnunet/notebooks/idc_nlst_nnunet_and_bpr_infer.ipynb. The queries that that were used to perform the filtering of the relevant series are here: 3. NSCLC-Radiomics: https://github.com/ImagingDataCommons/nnU-Net-BPR-annotations/blob/main/common/queries/NSCLC_Radiomics_query.txt. 4. NLST: https://github.com/ImagingDataCommons/nnU-Net-BPR-annotations/blob/main/common/queries/NLST_query.txt.
