# Supplementary material for: Enrichment of lung cancer computed tomography collections with AI-derived annotations
Source: Sci Data. 2024 Jan 4;11:25. doi: 10.1038/s41597-023-02864-y (PMC10766991; doi:10.1038/s41597-023-02864-y)
Supplement: Supplementary file 1 — Supplementary Information [file 41597_2023_2864_MOESM1_ESM.pdf]

Supplementary Information

Figure S1 2
Table S1 4

Figure S1

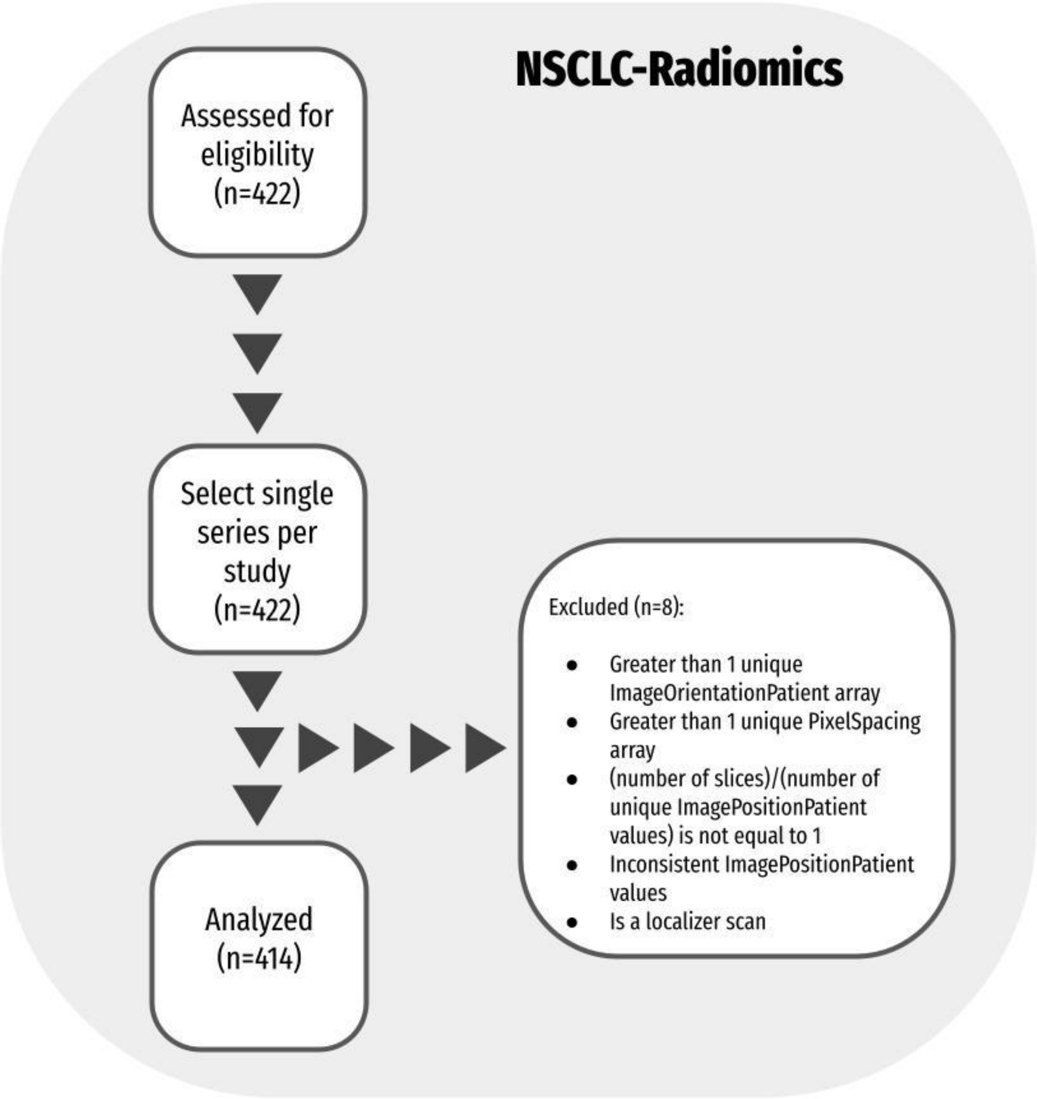

a) Filtering for the NSCLC-Radiomics collection DICOM CT series selected for the analysis.

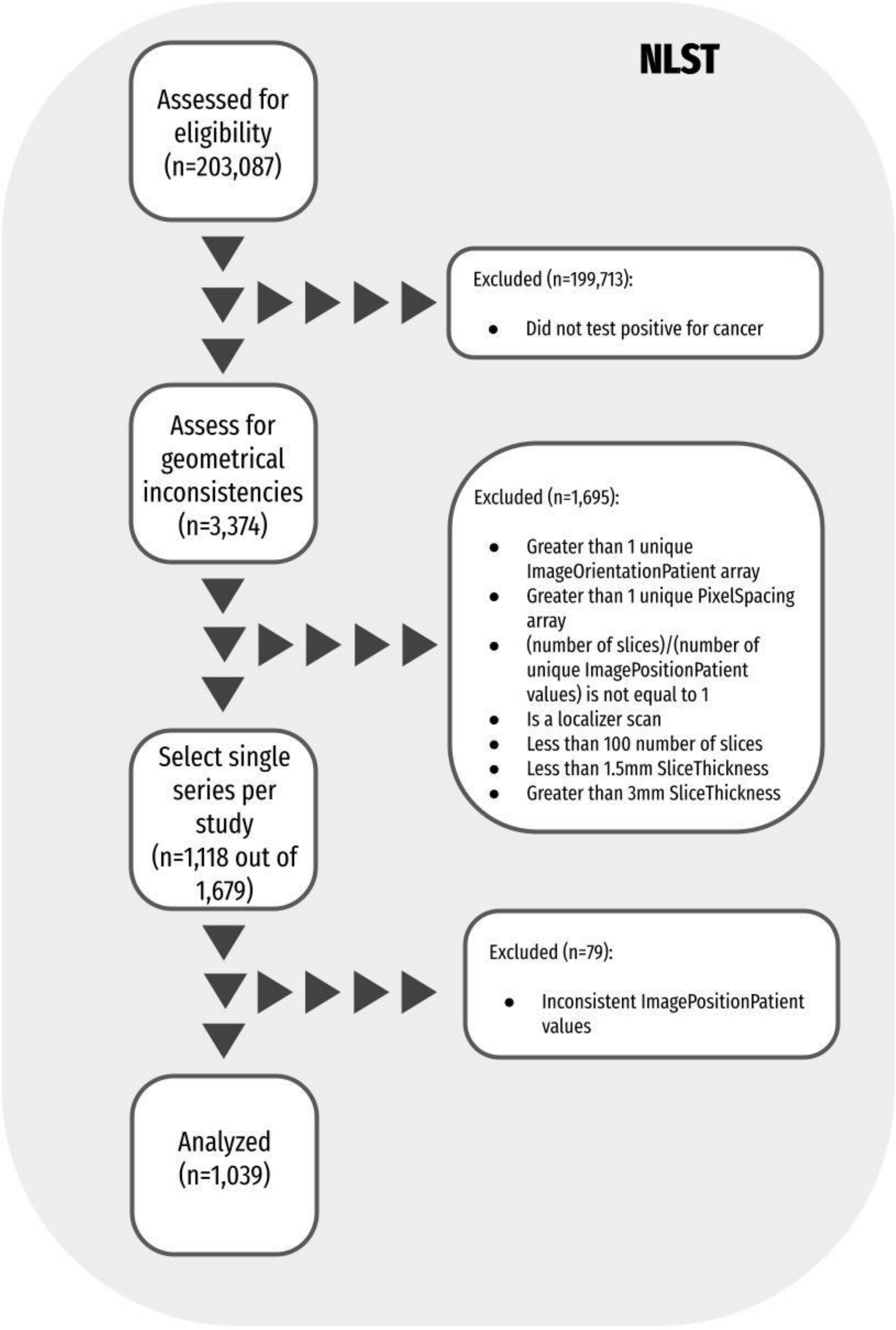

b) Filtering for the NLST collection DICOM CT series selected for the analysis.

Figure S1 - Overview of the selection process of series for the a) NSCLC-Radiomics collection, and the b) NLST collection.

Table S1

The landmarks produced from the body part prediction neural network were saved as DICOM Structured Reports, where a single report is saved for each series analyzed. The highdicom package[24] was used to generate the SR. The table below holds the information concerning the mapping of each of the body part regression codes to specific bones and landmarks. The optional modifier holds information about the location of the particular anatomical landmark. The table is also available here [https://github.com/ImagingDataCommons/nnU-Net-BPR-annotations/blob/main/bpr/data/bpr_landmarks_code_mapping.csv]. The list of landmarks was retrieved from the landmark look up table.[8]

BPR_code	CodingScheme Designator	CodeValue	CodeMeaning	modifier_ CodingScheme Designator	modifier_ CodeValue	modifier_ CodeMeaning
C1	SCT	14806007	C1 vertebra	DCM	111010	Center
C2	SCT	39976000	C2 vertebra	DCM	111010	Center
C3	SCT	113205007	C3 vertebra	DCM	111010	Center
C4	SCT	5329002	C4 vertebra	DCM	111010	Center
C5	SCT	36978003	C5 vertebra	DCM	111010	Center
C6	SCT	36054005	C6 vertebra	DCM	111010	Center
C7	SCT	87391001	C7 vertebra	DCM	111010	Center
Th1	SCT	64864005	T1 vertebra	DCM	111010	Center
Th2	SCT	53733008	T2 vertebra	DCM	111010	Center
Th3	SCT	1626008	T3 vertebra	DCM	111010	Center
Th4	SCT	73071006	T4 vertebra	DCM	111010	Center
Th5	SCT	56401006	T5 vertebra	DCM	111010	Center
Th6	SCT	45296009	T6 vertebra	DCM	111010	Center
Th7	SCT	62487009	T7 vertebra	DCM	111010	Center
Th8	SCT	11068009	T8 vertebra	DCM	111010	Center
Th9	SCT	82687006	T9 vertebra	DCM	111010	Center

Th10	SCT	7610001	T10 vertebra	DCM	111010	Center
Th11	SCT	12989004	T11 vertebra	DCM	111010	Center
Th12	SCT	23215003	T12 vertebra	DCM	111010	Center
L1	SCT	66794005	L1 vertebra	DCM	111010	Center
L2	SCT	14293000	L2 vertebra	DCM	111010	Center
L3	SCT	36470004	L3 vertebra	DCM	111010	Center
L4	SCT	11994002	L4 vertebra	DCM	111010	Center
L5	SCT	49668003	L5 vertebra	DCM	111010	Center
pelvis_start	SCT	8106092008	Pelvis	SCT	42161009	Bottom
femur_end	SCT	71341001	Femur	SCT	421812003	Top
pelvis_end	SCT	8106092008	Pelvis	SCT	421812003	Top
kidney	SCT	64033007	Kidney	None	None	None
lung_start	SCT	39607008	Lung	SCT	42161009	Bottom
lung_end	SCT	39607008	Lung	SCT	421812003	Top
liver_end	SCT	10200004	Liver	SCT	421812003	Top
teeth	SCT	28035005	Teeth, gums and supporting structures	DCM	111010	Center
nose	SCT	45206002	Nose	SCT	42161009	Bottom
eyes_end	SCT	81745001	Eye	SCT	421812003	Top
head_end	SCT	69536005	Head	SCT	421812003	Top

Table S1 - The landmark code mapping file used to create the TID1500 DICOM Structured Reports
